# Supplementary material for: Spatially resolved integrative analysis of transcriptomic and metabolomic changes in tissue injury studies
Source: Nat Commun. 2026 Jan 7;17:205. doi: 10.1038/s41467-025-68003-w (PMC12780049; doi:10.1038/s41467-025-68003-w)
Supplement: Supplementary file 1 — Supplementary Information [file 41467_2025_68003_MOESM1_ESM.pdf]

Supplementary Figures

Supplementary Figure 1

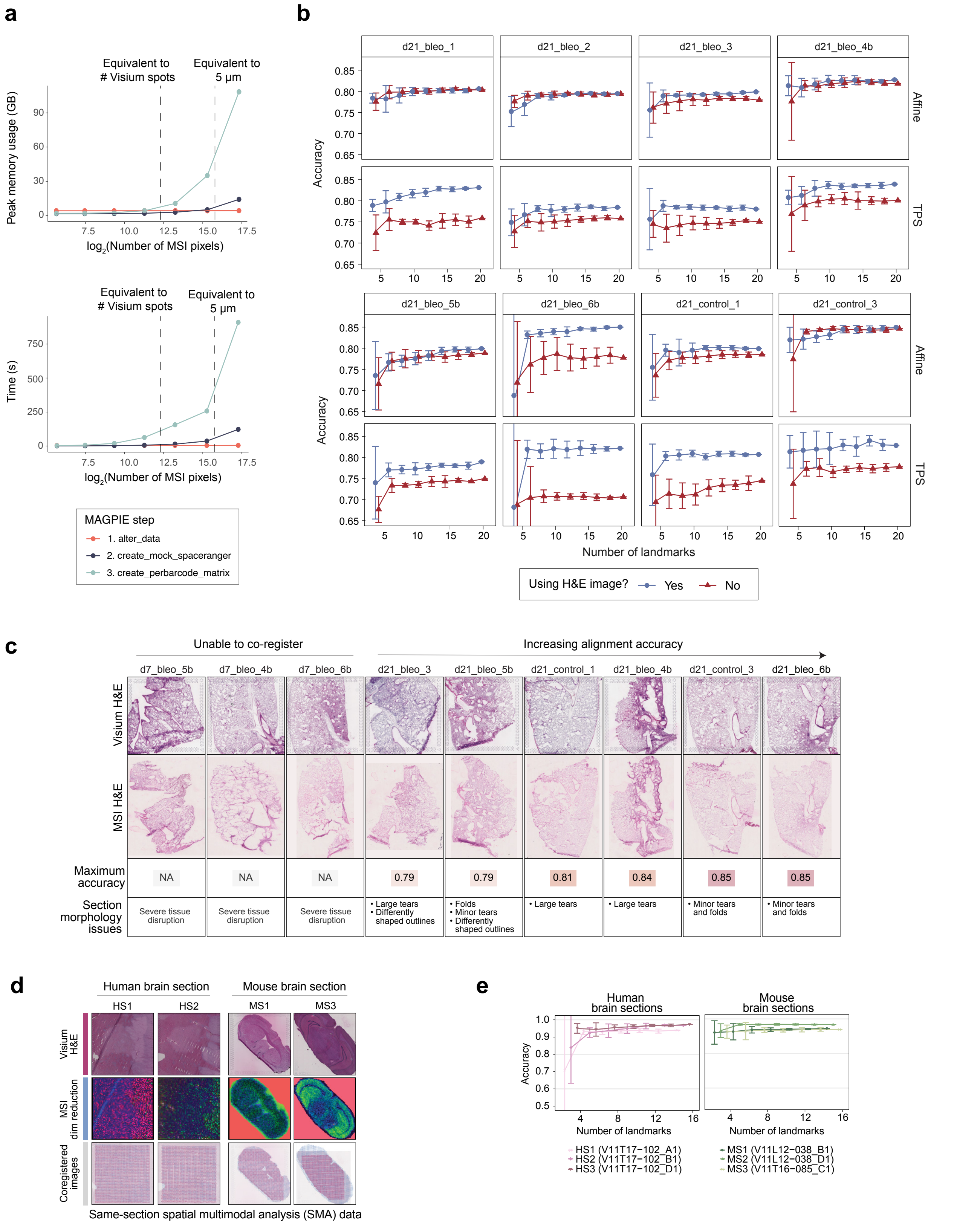

Supplementary Figure 1 | Further assessment and benchmarking of the flexibility and robustness of the MAGPIE integration pipeline. a, Assessment of peak memory usage and run-time across simulated varying MSI resolution and scale, per MAGPIE pipeline step (shown through colour). Vertical guidelines show equivalent number of pixels to number of Visium spots and to 5 µm resolution. **b,** Evaluation of varying *MAGPIE* hyperparameters in the BLM mouse model dataset showing alignment accuracy scores of tissue-to-background matching between Visium and MSI data after co-registration across a range of landmarks, employing a linear (affine) and non-linear (TPS) transform, and with or without using an intermediate MSI H&E image to assist with co-registration. Mean and 95% confidence intervals of accuracy (y-axis) are shown across 5 repeats for 4-20 landmarks (x-axis) and the use of an intermediate MSI H&E image is shown through the colour and shape. **c,** Ranking of the BLM mouse model samples by similarity (maximum accuracy measurement) between Visium and MSI sections comments on tissue section morphology artefacts that may influence alignment accuracy. Scale bars reflect 500 µm. We saw good performance across the 8 samples we were able to test the pipeline on, while 3 samples could not be coregistered due to tissue disruption. **d,** Illustration of flexibility of MAGPIE across different species and tissue types by showcasing its application to all samples in same-section Visium and MSI data from mouse and human brain tissue, followed by co-registered coordinates from both modalities. 6 samples were tested across human and mouse, all showing good alignment performance. Scale bars reflect 500 µm. **e,** Variation in accuracy of tissue against background matching between Visium and MSI data after co-registration (no intermediate MSI image provided) across a range of landmarks (3-15) with 5 repeats each. Error bars correspond to 95% confidence intervals, centred on the mean.

Supplementary Figure 2

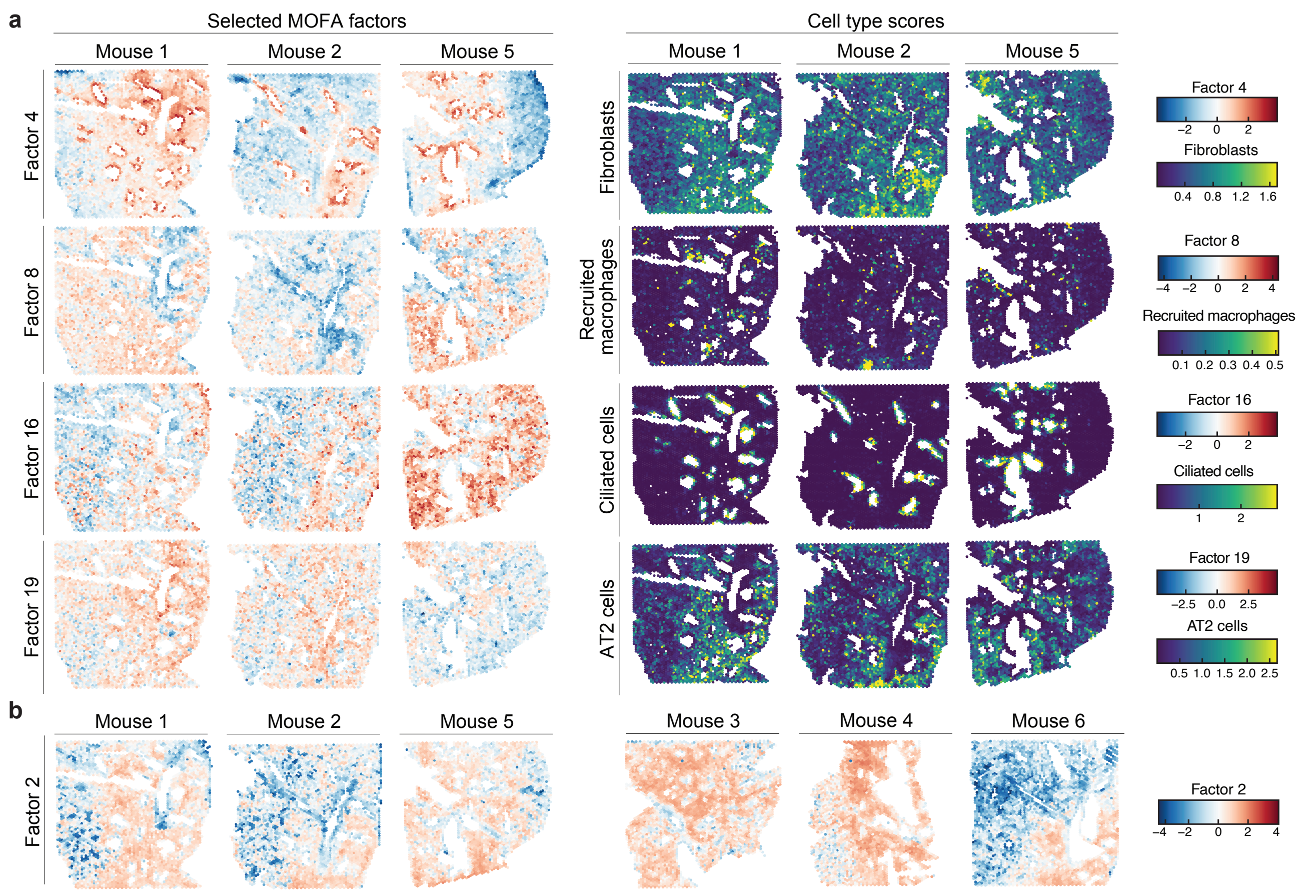

Supplementary Figure 2 | Spatial distribution of MOFA factors and key cell types in a bleomycin-treated mouse model. a, Spatial distributions of selected MOFA factors that showed contribution from both modalities (left) and key cell types which showed correlation with the shown MOFA factors and with MOFA Factor 2 (right, capped at 99th percentile). **b,** Spatial plots of the activity of MOFA Factor 2 across all 6 included samples.

Supplementary Figure 3

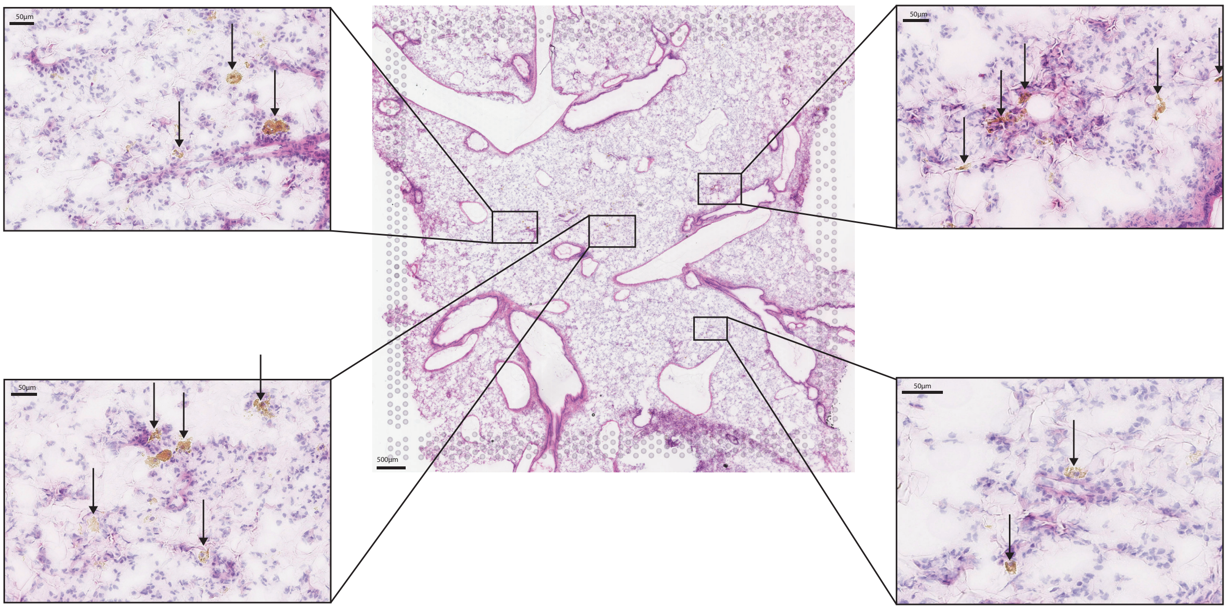

Supplementary Figure 3. Detection of brown-coloured crystal-like structures (black arrows), co-localising with dense AZX deposits, in a compound-treated rat lung sample, as seen in the H&E-stained Visium sections. Centre image scale bar indicates 500 µm. Scale bars in the zoomed-in inserts measure 50 µm. Only one sample was analysed in this case as a small case study.

Supplementary Figure 4

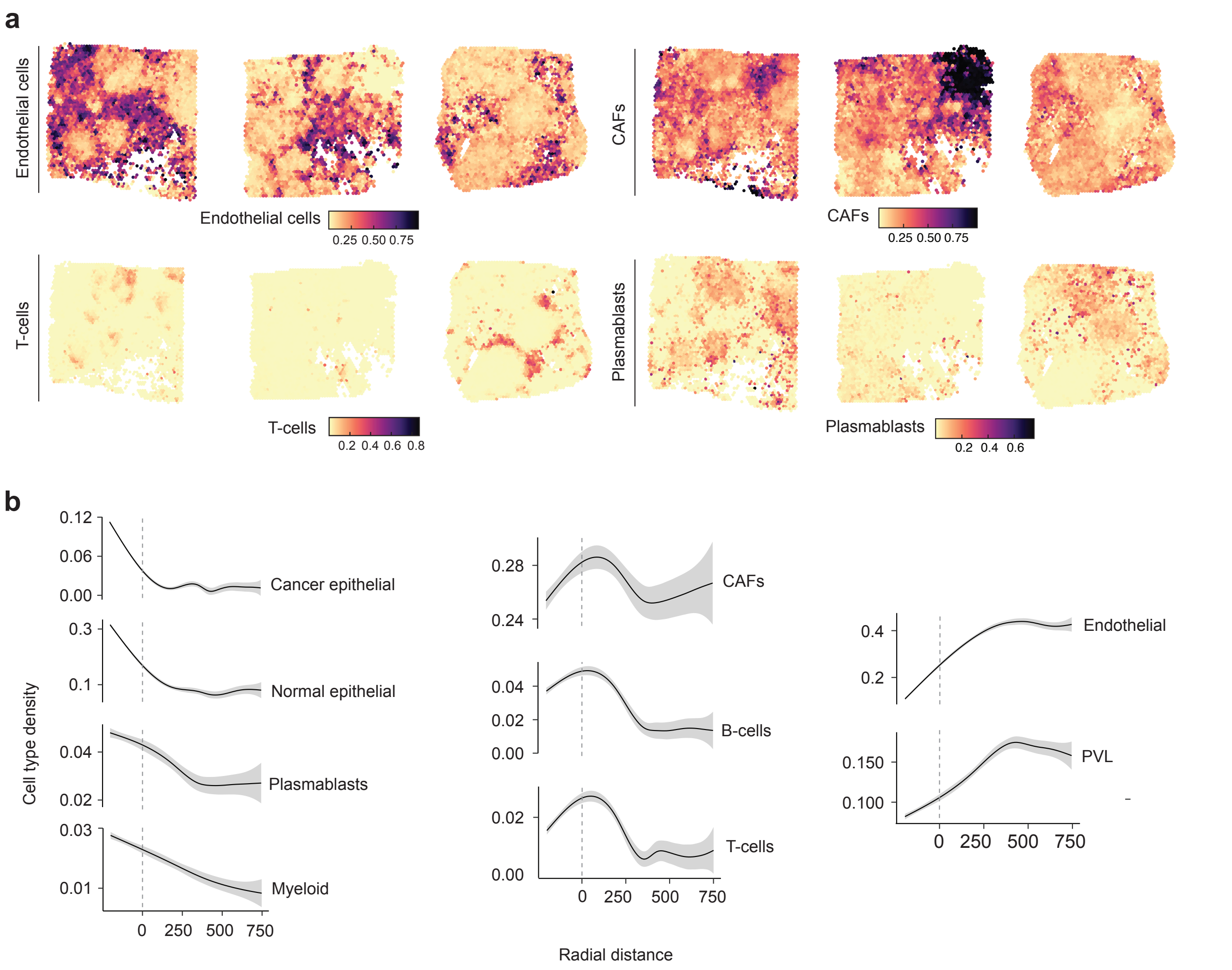

Supplementary Figure 4 | Further analysis of tumour microenvironment in human breast cancer through MAGPIE. **a,** Spatial distribution of cell type deconvolution scores (using CARD[1] with single-cell reference from Wu et al[2]), showing endothelial cells, T-cells, cancer-associated fibroblasts (CAFs) and plasmablasts. **b,** Line plot showing cell type densities varying across radial distance, smoothed using a generalised additive model with 95% confidence bands.

Supplementary Notes

Supplementary note 1: Lung tissue inflation and sample processing for spatial multimodal analysis

Preserving the native anatomical structure of lung tissue is essential for accurate spatial analysis[3]. Due to the highly elastic and air-filled nature of pulmonary tissue, deflation and collapse of alveolar spaces readily occur upon excision or during freezing; this process can compromise both histological integrity and molecular resolution. Therefore, controlled inflation is a standard procedure in pulmonary research, including for routine histological assessment such as H&E staining.

For the lung datasets used to illustrate the MAGPIE framework, we applied gentle inflation using a low-melting point agarose solution prior to snap freezing of mouse and rat lung tissue. This strategy ensures preservation of the alveolar architecture and spatial relationships between key structures such as bronchioles, vasculature, and interstitial compartments. Crucially, the inflation medium was chosen with downstream multimodal omics in mind. While the OCT (optimal cutting temperature) compound is commonly used for cryopreservation in Visium spatial transcriptomics workflows, it is known to interfere with mass spectrometry imaging (MSI) and can significantly impair metabolite detection. By contrast, low-melting point agarose is compatible with both transcriptomic and metabolomic analysis and introduces minimal interference in MSI workflows[4].

The agarose inflation protocol was tailored for each rodent model to match anatomical and physiological differences. For mouse lungs, ~0.4-0.5 mL of warm agarose solution was administered via tracheal instillation after perfusion with saline, and the tissue was snap-frozen directly without embedding. For rat lungs, a more extensive inflation volume (~4.5 mL) was used, followed by incubation on ice to allow gelation prior to rapid freezing in pre-chilled isopentane.

We emphasize that this approach is not a requirement of the MAGPIE computational framework; nor a generic requirement for biological experiments used for generating data subsequently processed using MAGPIE. The MAGPIE framework is compatible with spatial transcriptomics and metabolomics datasets derived from a variety of tissue types and preparation protocols. Agarose inflation was selected in this study, specifically for lung samples, to maximize structural integrity and compatibility across omics modalities. For other organs with denser or more self-supporting parenchyma, standard embedding or freezing protocols can be used.

Supplementary note 2: Resolution disparity handling in MAGPIE

As an optional final step to the MAGPIE snakemake pipeline, transformed MSI pixels can be aggregated to yield matching observations to Visium data, labelled by Visium barcodes. While Visium resolution is fixed at 55 µm, MSI resolution can vary more widely, from ~5 µm up to ~100 µm depending on the technology used. Another key difference between the two modalities is that Visium spots are spaced with 100 µm between the spot centres, while MSI pixels form a continuous grid across the tissue. To match the varied resolution options and potential different analysis focuses of our users, we provide several options for (1) which pixels are selected for aggregation to each Visium spot and (2) how the selected pixels are mathematically aggregated to spots.

To select which MSI pixels are aggregated for each Visium spot, users can choose between several options (**Supplementary Fig. 5**):

a) All pixels whose centres are within the true Visium radius of 55 µm
b) All pixels whose centres are within an expanded Visium radius, maximally extended to remove gaps between spots
c) All pixels with any overlap with the true Visium spot, calculated by estimating the MSI pixel size after transformation and adding the true Visium radius

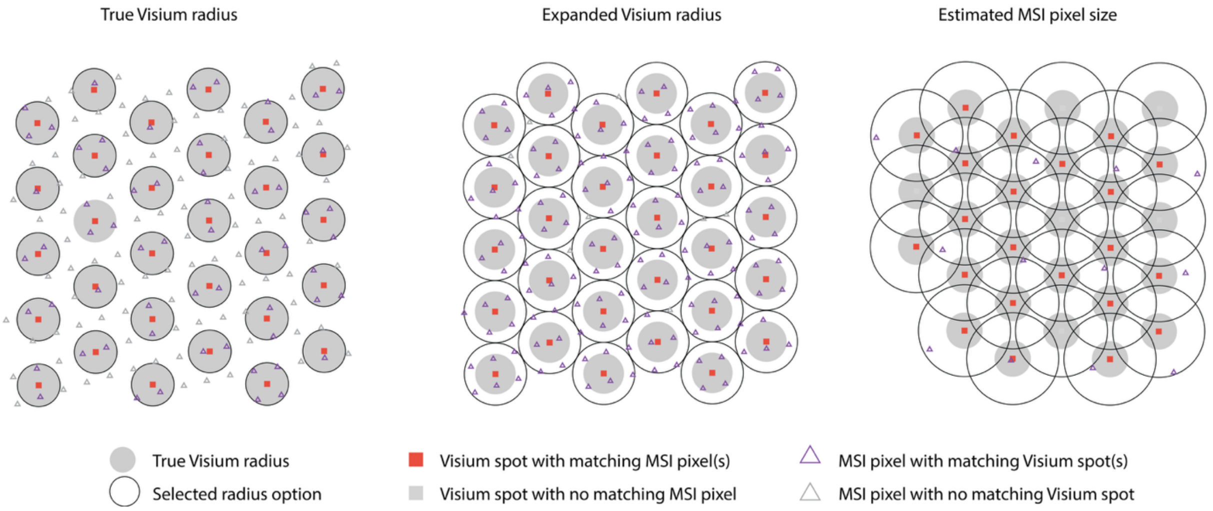

Supplementary Figure 5 | ***Visual summary of the different options for selecting which MSI pixels are assigned to which Visium spots within MAGPIE.*** *The choices shown here are a) all pixels whose centres fall within the true Visium radius of 55 µm (left), b) all pixels whose centres fall with expanded Visium radius, maximally expanded to remove gaps between spots (middle) and c) all pixels with any overlap with the true Visium spot, calculated by estimating the MSI pixel size after transformation and adding the true Visium radius (right). The summarisation can be performed using the mean or sum of intensities across pixels, or using the weighted average based on the distance from the Visium spot centre.*

The default option is the expanded Visium radius (option b); however, the true Visium radius (option a) may be a cleaner, more robust choice in the case of a very high resolution MSI dataset since there could be entire pixels which fall outside the true Visium radius, and these should therefore not be used in the aggregation. Alternatively, for MSI datasets of lower resolution than Visium, the user may choose to use the estimated MSI pixel size (option c) instead, as each pixel will contribute to multiple Visium spots; this approach will avoid gaps in coverage where there are Visium spots which overlap with MSI pixels but where the MSI pixel centre lies outside the spot and so the default setting would not link the MSI data for this pixel to the Visium spot.

To then aggregate the selected pixels, the current options are:

a) Mean, taking the mean intensity across all selected pixels, per peak
b) Sum, taking the sum of all intensities across all selected pixels, per peak
c) Weighted average of peak intensities, based on distance of selected pixels from the Visium spot centre

For each peak, the signal per Visium spot is calculated based on the user's choice; the aggregated expression levels are saved in Space Ranger-style format for further analysis.

Here, we summarise the number of pixels contributing to each Visium spot using a few examples (**Supplementary Fig. 6**)

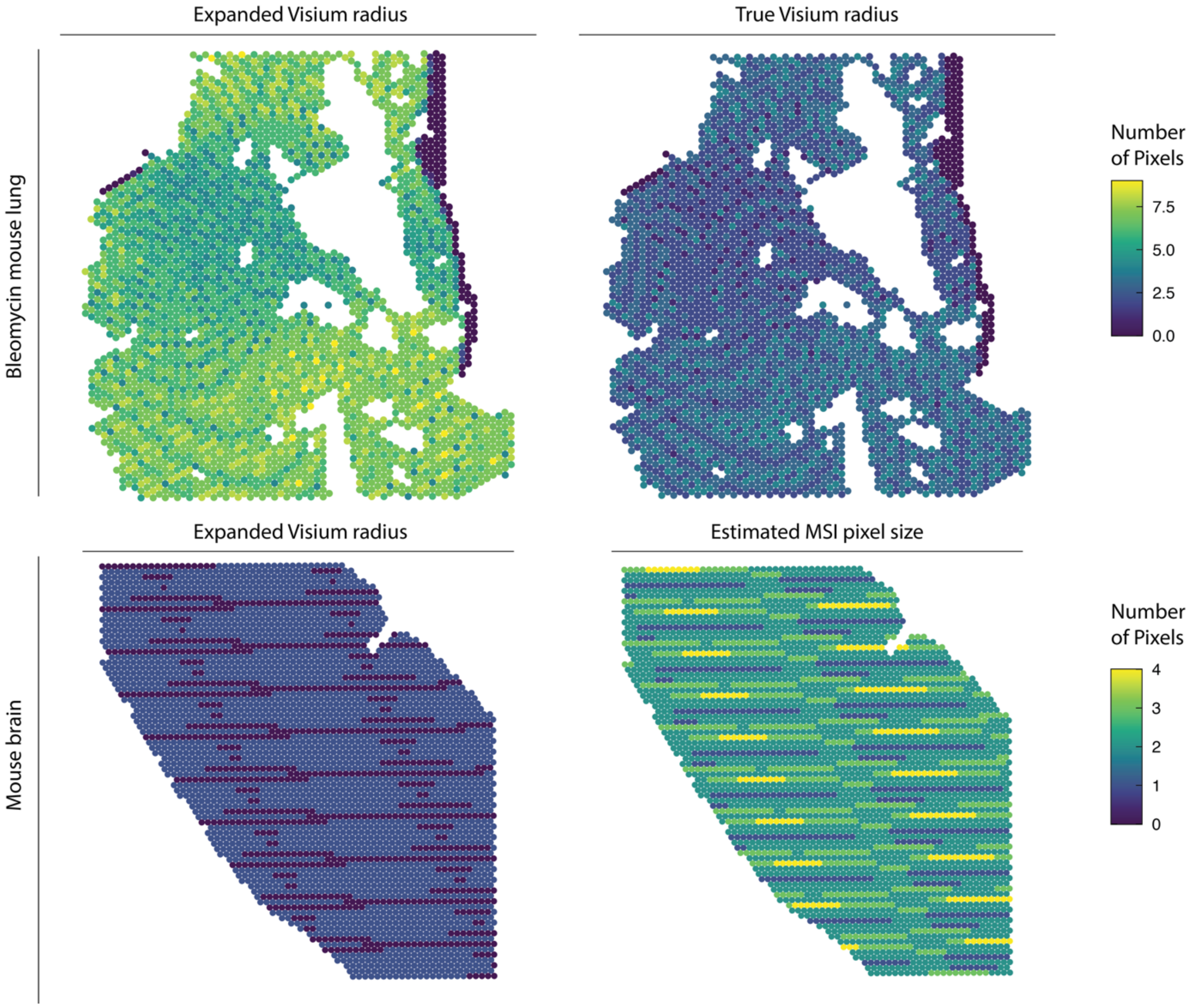

Supplementary Figure 6 | ***Examples of the number of MSI pixels aggregated to each Visium spot using different settings on a bleomycin mouse lung example*** *where MSI resolution is higher than Visium resolution (top)* ***and mouse brain example*** *which MSI resolution is lower than Visium resolution (bottom). In the mouse brain example, the offset of MSI pixels and Visium spots lead to horizontal patterns in space in the number of pixels per Visium spot. This is an example of a use case where a weighted average aggregation approach may produce more meaningful results than a standard average as it will better capture the relative contribution of each pixel.*

Supplementary note 3: Visualisation of brain region-specific spatial patterns

We illustrate the MAGPIE framework's ability to resolve highly spatially localised brain features using a same-section MALDI/Visium mouse brain sample (MS3 shown in the main manuscript, **Fig. 2f, Supplementary Fig. 1d-e**) from Vicari et al[5]. For this sample several small molecules were identified via MALDI-tandem MS (MS/MS)[5]. In **Supplementary Fig. 7**, we show examples of spatially localised features, specifically small molecules, genes and cell type scores (as made available in the original publication, Vicari et al (2024)[5]) corresponding to well-defined anatomical subregions:

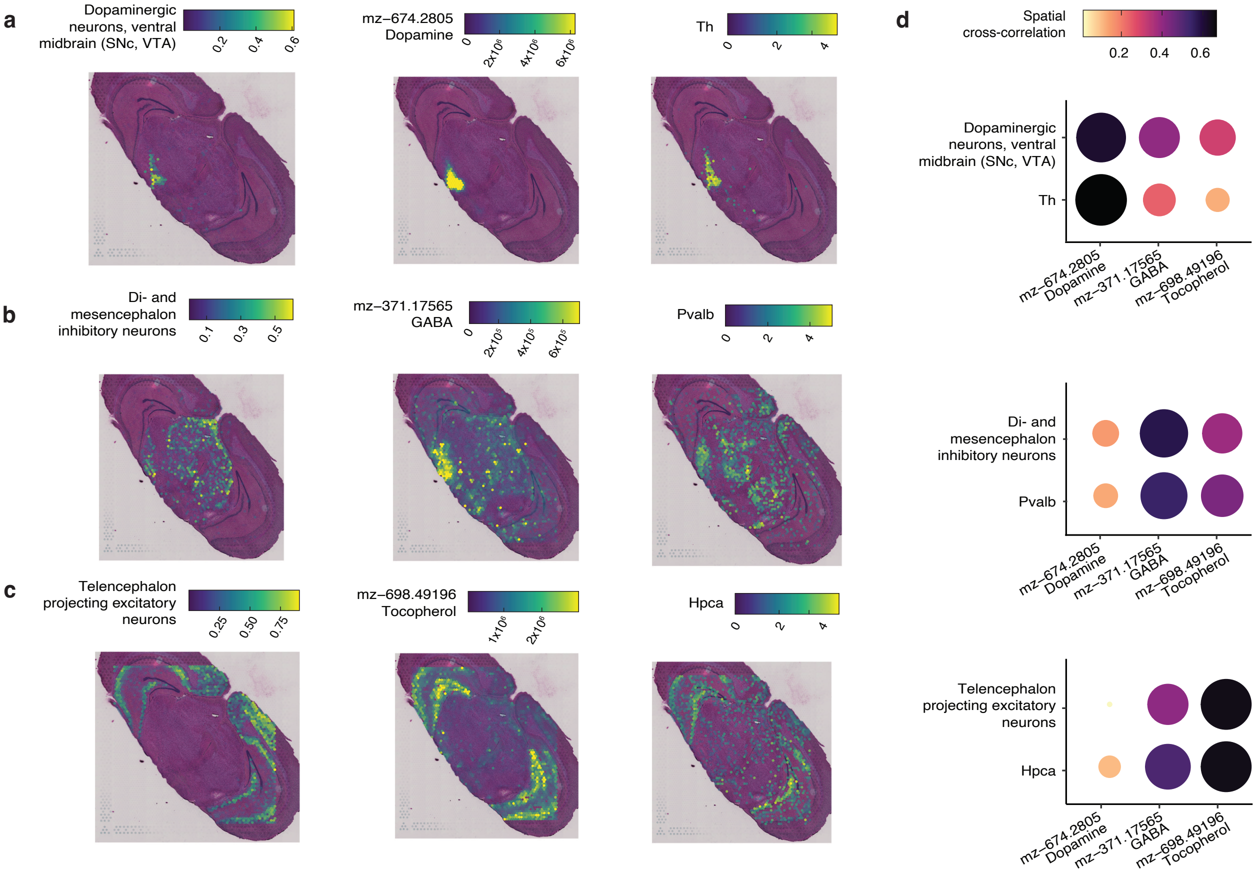

Supplementary Figure 7 | ***Illustration of MAGPIE's ability to resolve brain region-specific spatial patterns a-c****, Spatial distribution of (left) cell type scores, (middle) MSI peak intensities (capped at 99th percentile) and (right) gene expression for features localised to the (**a**) substantia nigra pars compacta (SNpc), (**b**) substantia nigra pars reticulata (SNr) and (**c**) CA1 and CA3 stratum pyramidale hippocampal subfields overlayed on H&E images. **d**, Dot plot of spatial cross-correlation between MSI peaks and cell type scores and gene expression. Both size and colour depict the cross-correlation coefficients.*

The substantia nigra is composed of two closely located but functionally distinct nuclei: the pars compacta (SNpc), which contains dopaminergic neurons, and the pars reticulata (SNr), characterised by GABAergic projection neurons. Within the SNpc, MAGPIE reveals, in a data-driven manner, a tight co-localisation of dopamine, tyrosine hydroxylase (*Th*) expression, and dopaminergic neuron scores (**Supplementary Fig. 7a**), consistent with *Th*'s role in dopamine biosynthesis and the established localisation of dopaminergic neurons. In the SNr, adjacent to the SNpc, MAGPIE highlights high GABA intensity overlapping with GABAergic neuron projection marker *Pvalb*, and transcriptional signatures of inhibitory neurons (**Supplementary Fig. 7b**). MAGPIE's ability to separate these two molecularly

and functionally distinct populations within such a compact anatomical region underlines its capacity to align molecular and morphological signals, even at subregional resolution.

Additionally, tocopherol (vitamin E), which plays a pivotal role in the nervous system and influences neuronal function, shows a distinct biodistribution, with high signal localised to several hippocampal structures including the CA1 and CA3 stratum pyramidale, fine-grained and morphologically distinct hippocampal structures (**Supplementary Figure 7c**). This is confirmed by H&E and high spatial cross-correlation with excitatory neuron scores and the known hippocampal marker *Hpca* (**Supplementary Figure 7d**). The accurate spatial mapping of metabolites, cell type scores and morphology within this compact region underscores MAGPIE's ability to isolate small, morphologically complex brain structures and potentially unveil novel connections between omics layers.

This case study highlights the versatility and efficiency of MAGPIE to meaningfully integrate transcriptomic, metabolomic, and cell type data to characterise functionally and anatomically relevant genes and metabolites (**Supplementary Figure 7d**), reflecting complex cytoarchitecture and within the resolution limitations of Visium. High-resolution methods like STEREO-seq[6] offer unique advantages in resolving very small structures, and co-registration approaches like MAGPIE cannot overcome limitations introduced by the underlying technologies or data types used as its input (Visium and MSI). However, MAGPIE provides a scalable, multi-modal approach that can deconvolve fine-grained spatial heterogeneity despite the inherent resolution limits of the input data.

Supplementary note 4: Limitations of Visium and MSI

We acknowledge that there are some limitations to the analysis and biological insights that can be formed on spatial multi-omics datasets. Firstly, spatial data, as used as input in MAGPIE, is inherently static, by focusing on tissue sections captured at a single time point. Assessments of causal inference to understand whether identified metabolic changes (MSI) are driving gene expression (Visium) and vice versa are beyond the scope of the current framework. A related challenge is posed by the difference in turnover rates between metabolites and transcripts, from seconds to hours. Metabolite levels may fluctuate rapidly in response to the tissue environment, while corresponding transcriptional changes may happen at a slower rate, such that transcriptional changes can be observed after the metabolite level has already returned to baseline[7]; by capturing static measurements we may see only the metabolite peak or only the transcriptional change persisting after the metabolic signal is gone, which may lead to misleading conclusions around regulatory relationships and spatial co-localisation of genes and metabolites. The interpretation can also be confounded by the inherent complexity of biological processes with individual metabolites playing roles in multiple pathways; if gene expression changes are offset from metabolic changes, then it is difficult to pinpoint the most biologically relevant pathway for the process of interest.

These challenges are inherent to measuring multiple molecules simultaneously[8,9]. However, the versatility and adaptability of MAGPIE can enable analysis of even more complex datasets involving additional imaging technologies, metabolite labelling, time series, or analyses using causal and dynamic models, further improving the confidence in inferring likely causal biological relationships and in prioritizing gene-metabolite pairs for follow up experiments.

Further validation of spot-level findings could be achieved through laser-capture microdissection coupled with LC-MS or RNA-seq, or by using (targeted) single-cell resolution spatial technologies such as Xenium. These approaches can provide valuable means to increase confidence in observed spatial relationships, specifically when used to complement Visium/MSI data. Considering the broader spatial context and whole-transcriptome coverage offered by Visium/MSI, combined with MAGPIE processing, remains essential for intact-tissue level analysis and spatial mapping of molecular interactions in health and disease.

Technical variability and batch effects, both across different MSI platforms and between separate MSI experiments[10,11], also represent significant challenges for the MSI community, as these factors can markedly influence the interpretation of outputs generated by MAGPIE. A critical consideration in mitigating such variability is rigorous control of sample preparation. All stages, from tissue preparation (e.g., fresh-frozen processing), embedding techniques designed to minimize variability[12], and sectioning, to subsequent vacuum packing and storage at −80°C[13], must be carefully standardized in accordance with recent literature. From the perspective of mass spectrometry methodology, it is essential to recognize that the ionization efficiency of specific metabolite classes may differ between methodologies, such as MALDI and DESI. These differences can impact the identification of metabolic pathways and the elucidation of the associated biological processes in MSI experiments. Notably, such effects may be further modulated by the selection of ionization polarity and, in the case of MALDI-MSI, by the choice of matrix. Therefore, the selection of the ionization source, MALDI matrix, and polarity should be tailored to the characteristics of the sample under investigation, with careful consideration given to the inherent limitations of each MSI platform[14]. On the data analysis front, we have illustrated the platform-agnostic applicability of MAGPIE by successfully processing both MALDI and DESI MSI datasets, derived by instruments from multiple providers.

As we have shown in several case studies, histology plays a valuable role in combination with SRT and MSI by providing essential structural context for analyses. As the field of spatial omics moves towards establishing a stronger link to histological assessment, preserving tissue morphology and

avoiding freezing artefacts becomes critical for accurate pathological interpretation. In this context, formalin-fixed paraffin-embedded (FFPE) tissue samples offer an attractive alternative to fresh frozen tissue. While workflows for Visium and other SRT methodologies have been established for FFPE tissues, integrating MSI with FFPE samples requires careful consideration of sample preparation protocols to ensure preservation of chemical identity and spatial localisation, particularly for smaller metabolites[15-18]. However, fresh frozen tissue can be preferable to FFPE due to concerns over RNA fragmentation and loss of transcript detection sensitivity caused by the formalin fixation and paraffin embedding process, as well as the more complex sample preparation required. As these protocols continue to develop, we expect that spatial multimodal datasets using both fresh frozen and fixed tissues will become increasingly important for spatial studies investigating molecular mechanisms in tissue biology, Due to the flexibility of the pipeline, we anticipate that the *MAGPIE* workflow will be adaptable to datasets produced with different tissue preparation methods and Visium platforms.

While current spatial multi-omics analysis still faces challenges such as those discussed around static measurements, resolution limitations, technical variability and sample preparation requirements, MAGPIE is able to accommodate diverse MSI technologies and provides a flexible workflow to enable cross-platform integration, which could lay the groundwork for future studies moving beyond static snapshots and leading to capturing more dynamic, higher resolution views of tissue biology.

Supplementary References

1 Ma, Y., Zhou, X., Ma, Y. & Zhou, X. Spatially informed cell-type deconvolution for spatial transcriptomics. *Nature Biotechnology 2022 40:9* **40** (2022-05-02). https://doi.org/10.1038/s41587-022-01273-7

2 Wu, S. Z. *et al.* A single-cell and spatially resolved atlas of human breast cancers. *Nature Genetics 2021 53:9* **53** (2021-09-06). https://doi.org/10.1038/s41588-021-00911-1

3 Braber, S., Verheijden, K. A. T., Henricks, P. A. J., Kraneveld, A. D. & Folkerts, G. A comparison of fixation methods on lung morphology in a murine model of emphysema. *American Journal of Physiology-Lung Cellular and Molecular Physiology* **299** (2010). https://doi.org/10.1152/ajplung.00192.2010

4 Dannhorn, A. *et al.* Universal sample preparation unlocking multimodal molecular tissue imaging. *Analytical Chemistry* (2020). https://doi.org/10.1021/acs.analchem.0c00826

5 Vicari, M. *et al.* Spatial multimodal analysis of transcriptomes and metabolomes in tissues. *Nature Biotechnology* (2024). https://doi.org/10.1038/s41587-023-01937-y

6 Chen, A. *et al.* Spatiotemporal transcriptomic atlas of mouse organogenesis using DNA nanoball-patterned arrays. *Cell* **185**, 1777-1792 e1721 (2022). https://doi.org/10.1016/j.cell.2022.04.003

7 Dong, H. *et al.* Metabolic memory: mechanisms and diseases. *Signal Transduction and Targeted Therapy 2024 9:1* **9** (2024-02-28). https://doi.org/10.1038/s41392-024-01755-x

8 Lähnemann, D. *et al.* Eleven grand challenges in single-cell data science. *Genome Biology 2020 21:1* **21** (2020-02-07). https://doi.org/10.1186/s13059-020-1926-6

9 Canzler, S. *et al.* Prospects and challenges of multi-omics data integration in toxicology. *Archives of Toxicology 2020 94:2* **94** (2020-02-08). https://doi.org/10.1007/s00204-020-02656-y

10 Balluff, B., Hopf, C., Siegel, T. P., Grabsch, H. I. & Heeren, R. M. A. Batch Effects in MALDI Mass Spectrometry Imaging. *Journal of the American Society for Mass Spectrometry* **32** (2021 Feb 1). https://doi.org/10.1021/jasms.0c00393

11 Boskamp, T. *et al.* Cross-Normalization of MALDI Mass Spectrometry Imaging Data Improves Site-to-Site Reproducibility. *Anal Chem* **93**, 10584-10592 (2021). https://doi.org/10.1021/acs.analchem.1c01792

12 Dannhorn, A. *et al.* Universal Sample Preparation Unlocking Multimodal Molecular Tissue Imaging. *Analytical Chemistry* **92** (June 10, 2020). https://doi.org/10.1021/acs.analchem.0c00826

13 Swales, J. G. *et al.* Quantitation of Endogenous Metabolites in Mouse Tumors Using Mass-Spectrometry Imaging - PubMed. *Analytical chemistry* **90** (05/15/2018). https://doi.org/10.1021/acs.analchem.7b05239

14 Saharuka, V. *et al.* Large-Scale Evaluation of Spatial Metabolomics Protocols and Technologies. *bioRxiv* (2024-01-31). https://doi.org/10.1101/2024.01.29.577354

15 Dannhorn, A. *et al.* Evaluation of Formalin-Fixed and FFPE Tissues for Spatially Resolved Metabolomics and Drug Distribution Studies. *Pharmaceuticals 2022, Vol. 15, Page 1307* **15** (2022-10-23). https://doi.org/10.3390/ph15111307

16 Aichler, M. *et al.* Molecular similarities and differences from human pulmonary fibrosis and corresponding mouse model: MALDI imaging mass spectrometry in comparative medicine. *Lab Invest* **98**, 141-149 (2018). https://doi.org/10.1038/labinvest.2017.110

17 Kreutzer, L. *et al.* Simultaneous metabolite MALDI-MSI, whole exome and transcriptome analysis from formalin-fixed paraffin-embedded tissue sections. *Lab Invest* **102**, 1400-1405 (2022). https://doi.org/10.1038/s41374-022-00829-0

18 Erlmeier, F. *et al.* Matrix-Assisted Laser Desorption/Ionization Mass Spectrometry Imaging: Diagnostic Pathways and Metabolites for Renal Tumor Entities. *Oncology* **101**, 126-133 (2023). https://doi.org/10.1159/000526436
