# Supplementary material for: Spatially resolved integrative analysis of transcriptomic and metabolomic changes in tissue injury studies
Source: Nat Commun. 2026 Jan 7;17:205. doi: 10.1038/s41467-025-68003-w (PMC12780049; doi:10.1038/s41467-025-68003-w)
Supplement: Supplementary file 2 — Reporting Summary [file 41467_2025_68003_MOESM2_ESM.pdf]

nature portfolio

Corresponding author(s): Irina Mohorianu
Jennifer Tan
Anna Ollerstam

Last updated by author(s): Dec 3, 2025

Reporting Summary

Nature Portfolio wishes to improve the reproducibility of the work that we publish. This form provides structure for consistency and transparency in reporting. For further information on Nature Portfolio policies, see our Editorial Policies and the Editorial Policy Checklist.

Statistics

For all statistical analyses, confirm that the following items are present in the figure legend, table legend, main text, or Methods section.

n/a	Confirmed	
☐	☒	The exact sample size (n) for each experimental group/condition, given as a discrete number and unit of measurement
☐	☒	A statement on whether measurements were taken from distinct samples or whether the same sample was measured repeatedly
☐	☒	The statistical test(s) used AND whether they are one- or two-sided<br>*Only common tests should be described solely by name; describe more complex techniques in the Methods section.*
☒	☐	A description of all covariates tested
☐	☒	A description of any assumptions or corrections, such as tests of normality and adjustment for multiple comparisons
☐	☒	A full description of the statistical parameters including central tendency (e.g. means) or other basic estimates (e.g. regression coefficient) AND variation (e.g. standard deviation) or associated estimates of uncertainty (e.g. confidence intervals)
☐	☒	For null hypothesis testing, the test statistic (e.g. F, t, r) with confidence intervals, effect sizes, degrees of freedom and P value noted<br>*Give P values as exact values whenever suitable.*
☒	☐	For Bayesian analysis, information on the choice of priors and Markov chain Monte Carlo settings
☒	☐	For hierarchical and complex designs, identification of the appropriate level for tests and full reporting of outcomes
☐	☒	Estimates of effect sizes (e.g. Cohen's d, Pearson's r), indicating how they were calculated

Our web collection on statistics for biologists contains articles on many of the points above.

Software and code

Policy information about availability of computer code

Data collection	For DESI MSI data acquisition, Omnispray 2D v2.0.1 (Prosolia, Indianapolis, USA), Xcalibur v 4.5.474.0 (Thermo Fisher Scientific Inc), MSConvert (ProteoWizard toolbox version 3.0.4043) and imzML converter v1.3 software were used.<br><br>For pathology assessment, Aperio CS2 digital pathology scanner (Aperio Tech., Oxford, UK), and QuPath 0.2366 were applied.<br><br>All Visium data in this study was processed from raw FASTQ files using Space Ranger 1.2.2 for mouse bleomycin samples or 1.3.1 for rat AZX samples (10x Genomics).
Data analysis	DESI MSI data was processed using SCiLS Lab v2023b and the SCiLS Lab API.<br><br>Co-registration using MAGPIE was performed with Python (v3.10.11) and the following Python modules:<br>snakemake==7.32.4<br>shiny==1.1.0<br>matplotlib==3.9.2<br>pandas==2.2.3<br>numpy==1.26.4<br>scikit-image==0.24.0<br>pathlib==1.0.1<br>scikit-learn==1.5.2<br>scipy==1.14.1

h5py==3.12.1
scanpy==1.14.1

Downstream analysis was performed using R (v4.3.0) and the following R packages:
semla v1.2.1
ggplot2 v3.4.2
tidyverse v2.2.0
ggpubr v0.6.0
patchwork v1.1.2
data.table v1.14.8
MOFA2 v1.10.0
GENIE3 v1.22.0
igraph v2.0.3
MERINGUE v1.0
gprofiler2 v0.2.1

The MAGPIE software is available at https://github.com/Core-Bioinformatics/magpie with a mirrored deposition to Zenodo under https://doi.org/10.5281/zenodo.17751881. Processed data, MAGPIE inputs and data analysis scripts can also be found on Zenodo under 10.5281/zenodo.17789448.

For manuscripts utilizing custom algorithms or software that are central to the research but not yet described in published literature, software must be made available to editors and reviewers. We strongly encourage code deposition in a community repository (e.g. GitHub). See the Nature Portfolio guidelines for submitting code & software for further information.

Data

Policy information about availability of data

All manuscripts must include a data availability statement. This statement should provide the following information, where applicable:

- Accession codes, unique identifiers, or web links for publicly available datasets
- A description of any restrictions on data availability
- For clinical datasets or third party data, please ensure that the statement adheres to our policy

Raw and processed RNA-sequencing data for bleomycin-treated mouse Visium samples are available on Gene Expression Omnibus (GEO) under accession GSE267904. DESI MSI data for the bleomycin-treated mouse lung samples are available on MetaboLights under accession MTBLS13445. Raw and processed RNA-sequencing data for the AZX-treated rat lung Visium samples are available on Gene Expression Omnibus (GEO) under accession GSE312168. DESI MSI data for AZX-treated rat lung sample are available on MetaboLights under accession MTBLS13445.

For the same-section Visium MALDI MSI multimodal datasets on human and mouse brain tissue, the datasets are available from Mendeley Data (DOI: 10.17632/w7nw4km7xd.1).

For the same-section Visium DESI MSI multimodal datasets on human breast cancer tissue, the datasets are available from Dataverse (DOI:10.7910/DVN/GZFCWC), reference number 107910.

Processed data for all datasets are available on Zenodo under accession 10.5281/zenodo.17789448.

Research involving human participants, their data, or biological material

Policy information about studies with human participants or human data. See also policy information about sex, gender (identity/presentation), and sexual orientation and race, ethnicity and racism.

Reporting on sex and gender	No participants have been recruited for this study. In the publicaly available human brain and breast cancer datasets, consistent with the original publications related to the datasets (doi: 10.1038/s41587-023-01937-y, 10.1002/anie.202502028), sex and gender were not considered important for the purposes of our methodological study.
Reporting on race, ethnicity, or other socially relevant groupings	No participants have been recruited for this study. As in the original publications where the datasets were made publicly available, race and ethnicity were not important for our methodological study.
Population characteristics	No participants have been recruited for this study. As in the original publication, population characteristics were not important for our methodological study. The human post-mortem sample used to generate the publicly available human brain dataset was obtained from the Harvard Brain Tissue Resource Center at the McLean Hospital, Belmont, MA, USA. The human sample was from the caudate-putamen of a man who died at 94 years of age. The neuropathological diagnosis was Parkinson's disease in Braak stage 3. The human breast cancer samples were obtained from NCI Cooperative Human Tissue Network (CHTN) from patients with invasive lobular breast cancer.
Recruitment	No participants have been recruited for this study. The post-mortem sample used to generate the publicly available human brain dataset was obtained from the Harvard Brain Tissue Resource Center at the McLean Hospital (Belmont, MA, USA) and the human breast cancer dataset tissue was obtained from the NCI Cooperative Human Tissue Network(CHTN).
Ethics oversight	No participants have been recruited for this study. The analyses performed in the original publication for the brain dataset was approved by the local ethical committee (Karolinska Institutet, Stockholm, Sweden, no 2014/1366-31).

Note that full information on the approval of the study protocol must also be provided in the manuscript.

Field-specific reporting

Please select the one below that is the best fit for your research. If you are not sure, read the appropriate sections before making your selection.

[x] Life sciences [ ] Behavioural & social sciences [ ] Ecological, evolutionary & environmental sciences

For a reference copy of the document with all sections, see nature.com/documents/nr-reporting-summary-flat.pdf

Life sciences study design

All studies must disclose on these points even when the disclosure is negative.

Sample size	The approach was tested on 4 datasets: [1] bleomycin-treated mouse samples (n=9), [2] AZX-treated rat lung sample (n=1) and [3] human (n=3), mouse (n=3) brain tissue and [4] human breast cancer tissue (n=3).<br><br>No sample size calculation was performed as this work focused on a new methodology and demonstrating its applicability on several different tissue types.
Data exclusions	Visium data was filtered using spot and gene-based thresholds as described in Methods. DESI MSI data was filtered using pixel-based and peak-based parameters described in Methods.
Replication	We benchmarked our approach on 2 datasets to assess the robustness and reproducibility of our results.
Randomization	In the full published bleomycin study, mice were randomly assigned to treatment groups to ensure that experimental conditions were evenly distrubuted. For the purposes of our methodology study we selected 11 of these animals. In the full AZX-dosed rat study, rats were randomly assigned to dose groups but we selected one sample for this methodology study.
Blinding	Our study does not involve group allocation that requires blinding.

Reporting for specific materials, systems and methods

We require information from authors about some types of materials, experimental systems and methods used in many studies. Here, indicate whether each material, system or method listed is relevant to your study. If you are not sure if a list item applies to your research, read the appropriate section before selecting a response.

Materials & experimental systems

n/a	Involved in the study	
x		Antibodies
x		Eukaryotic cell lines
x		Palaeontology and archaeology
	x	Animals and other organisms
x		Clinical data
x		Dual use research of concern
x		Plants

Methods

n/a	Involved in the study	
x		ChIP-seq
x		Flow cytometry
x		MRI-based neuroimaging

Animals and other research organisms

Policy information about studies involving animals; ARRIVE guidelines recommended for reporting animal research, and Sex and Gender in Research

Laboratory animals	For bleomycin-treated mouse dataset, C57BL/6NCrl mice (8 weeks of age upon arrival) were purchased from Charles River, Germany. Following a five-day acclimatization period, mice were subjected to bleomycin dissolved in saline or saline alone. Lung samples were collected on day 7 (d7) and day 21 (d21) post-bleomycin challenge.<br><br>For the AZX treatment dataset, the male Wistar Han rat (7 weeks old upon arrival and 10 weeks at the start of dosing) was purchased from Charles River, Germany. The rat was subjected to daily inhalation exposure via snout-only administration for 14 days, after which the lung sample was collected. After a five-day period of acclimatization to the vivarium, the rat was acclimated to the method of restraint in the inhalation system over a five-day period preceding the first exposure.
Wild animals	The study did not involve wild animals
Reporting on sex	All mice included in the bleomycin-treated mouse dataset (n=11) were female due to previously observed sex-based variation in the bleomycin model of pulmonary fibrosis and prior optimisation of the model for female mice at AstraZeneca R&D Gothenburg. The AZX-treated sample (n=1) came from a male rat.
Field-collected samples	The study did not include samples collected from the field.

Ethics oversight	Animal care and handling adhered to the standards established by the Council of Europe ETS123 AppA, the Helsinki Convention for the Use and Care of Animals, Swedish legislation, and AstraZeneca global internal standards. All experiments were ethically approved by the Gothenburg Ethics Committee for Experimental Animals in Sweden, complying with Directive 2010/63/EU. The studies received local Ethical committee approval in Gothenburg (EA000680-2017 and 2020-002853) with the assigned site number 31-5373/11.

Note that full information on the approval of the study protocol must also be provided in the manuscript.

Plants

Seed stocks	*Report on the source of all seed stocks or other plant material used. If applicable, state the seed stock centre and catalogue number. If plant specimens were collected from the field, describe the collection location, date and sampling procedures.*
Novel plant genotypes	*Describe the methods by which all novel plant genotypes were produced. This includes those generated by transgenic approaches, gene editing, chemical/radiation-based mutagenesis and hybridization. For transgenic lines, describe the transformation method, the number of independent lines analyzed and the generation upon which experiments were performed. For gene-edited lines, describe the editor used, the endogenous sequence targeted for editing, the targeting guide RNA sequence (if applicable) and how the editor was applied.*
Authentication	*Describe any authentication procedures for each seed stock used or novel genotype generated. Describe any experiments used to assess the effect of a mutation and, where applicable, how potential secondary effects (e.g. second site T-DNA insertions, mosiacism, off-target gene editing) were examined.*
