# Supplementary material for: Spatially resolved integrative analysis of transcriptomic and metabolomic changes in tissue injury studies
Source: Nat Commun. 2026 Jan 7;17:205. doi: 10.1038/s41467-025-68003-w (PMC12780049; doi:10.1038/s41467-025-68003-w)
Supplement: Supplementary file 3 — Description of Additional Supplementary Files [file 41467_2025_68003_MOESM3_ESM.pdf]

Supplementary Data 1: Lists of top genes and peaks contributing to the Visium and MSI modality respectively in each MOFA factor

Supplementary Data 2: Summary of nodes and edges in GENIE3-inferred network using top genes and peaks from MOFA Factor 2
